# Acknowledgement to Reviewers of *Pharmacy* in 2018

**DOI:** 10.3390/pharmacy7010008

**Published:** 2019-01-11

**Authors:** Pharmacy Editorial Office

**Affiliations:** MDPI, St. Alban-Anlage 66, 4052 Basel, Switzerland

Rigorous peer-review is the corner-stone of high-quality academic publishing. The editorial team greatly appreciates the reviewers who contributed their knowledge and expertise to the journal’s editorial process over the past 12 months. In 2018, a total of 131 papers were published in the journal, with a median time to first decision of 19 days and a median time to publication of 46 days. The editors would like to express their sincere gratitude to the following reviewers for their cooperation and dedication in 2018:

Adams, Katie SmithwickLee, Chooi YengAguero, Sandra M.Lee, JiehyunAhmed, ZehraLee, KennethAlessandri, ClaudiaLeon, Juan FranciscoAsatryan, BabkenLewis, SusanAshigbie, PaulLim, RosemaryAshish, PathakLinder, Stephen H.Aslani, ParisaLing, HuaAslanidis, TheodorosLondras, Fiona DeAssari, ShervinLongyhore, Daniel S.Austin, ZubinLouie, JessicaAxon, David RhysLow, AlanBabin, JenniferLu, JunBađun, MarijanaLuetsch, KarenBaker, DanialLuong, Duong (Donna)Barnard, MarieMadzhidova, ShirinBeckett, Emily AMandelbaum, DavidBelousova, VictoriaMantzourani, EfiBennett, James P.Manzo, CiroBennett, Jill HinesMartins, NatáliaBerry, TriciaMarvanova, MarketaBillups, SarahMathieson, StephanieBlack, Esther P.Maugeri, AndreaBliuc, DanaMayberry, KatelynnBockarie, AbuMcDermott, Michael T.Boggs, Douglas L.McDonagh, LorraineBožič, BorutMcGory, RobertBratt, AlisonMestres, ConxitaBrown, AlysonMihalopoulos, CleopatraBrown, Jacob T.Mikhailov, Theresa ABurghardt, KyleMira, José JoaquínButner, JennaMishra, ManishButterfoss, KirstenMittal, ManishCarter, StephenMixon, Amanda S.Carvajal, Manuel J.Morgan, JillCastellanos, DanielMorrison, JulietCavé, ChristianMorrissey, HanaCayón De Las Cuevas, JoaquínMotycka, CarolCerra, FrankMuceniece, RutaChaudhari, Shilpa PraveenMullen, Tyler RChin, PaulMűller, MirelaChng, Hui TingMunro, AlisonChoonara, ImtiMutie, MosesCooper, JasonNajib, Jadwiga S.Cornu, CatherineNeumann, TimCox, Anthony R.Newsome, Andrea S.Crawford, Stephanie Y.Nori, PriyaCurrie, KayNorman, Elizabeth J.Dang, DevraNormann, Sven A.De Voest, MargaretObreza, AlešDemirkol, ApoO’Dwyer, MáireDering, AlisonPammett, Robert T.Deshpande, MaithiliPapadakaki, MariaDhital, RanjitaParrish, RichardDickens, BernardPatel, NileshDilles, TinnePatka, John H.Druedahl, Louise C.Peña Quintana, LuisDuncan, PollyPerri III, MatthewDuong, PhuPeterson, GregoryEhret, Megan J.Petousis-Harris, Helen AspasiaEsteve-Romero, JosepPiper, BrianFejzić, JasminaPistelli, FrancescoFetterman, James W.Plummer, VirginiaFouladkhah, AliyarPorebski, GrzegorzFourtounas, CostasPotter, KathleenFousteris, ManolisProcyshyn, Ric M.Fox, CarolPuglisi-Weening, MelanyFrisk, PiaQiu, YangGallagher, PaulRamachandran, SujithGauld, Natalie JoanRascher, WolfgangGeraghty, PatrickRashid, HarunorGettleman, LawrenceRasiah, RohanGilissen, LiesbethRavula, AbhigyanGionfriddo, Michael RRichardson, Carolyn M.Głąbska, DominikaRobinson, DanielGlass, BeverleyRodis, Jennifer L.Gmeiner, TanjaRogers, GaryGoad, JeffRogobete, Alexandru FlorinGopalan, Rajendran CRogozea, LilianaGraudins, Linda V.Roque, FátimaGrieve, LorinRosenthal, MeaganGubin, DenisRubio-Valera, MariaGudka, SajniRutter, PaulGuirguis, AmiraRyder, NathanGunaldo, Tina PatelRyder, SheilaGupta, VasudhaSahm, Laura JaneGura, KathleenSandy, LarissaHan, JayoungSantulli, GaetanoHanna, Lezley-AnneSaurav, KumarHansford, KarlSeed, Sheila MHardisty, JessicaSerpa, SandroHaron, MonaSharma, PawanHarrison, ReemaShearer, BrennaHassan, AmanyShelton, Chasity M.Heslop, IanSherer, Jeffrey T.Higa, Gerald M.Siebers, RobHillman, AshleySingh, ShaktiHilton, Thomas F.Singh, SomnathHirasawa, NoriyasuSkoy, ElizabethHoldford, DavidSlack, MarionHolle, LisaSohn, MinjiHolman, Andrei CStehlik, PaulinaHoneywell, Marlon S.Stupans, IevaHoshiko, HeatherTaylor, DeniseHousman, Seth T.Taylor, Denise A.Huei Xin, LouTaylor, JeffHughes, JeffTeschke, RolfHughes, LouiseTook, RoxaneIbrahim, KindaTucker, StevenIorga, MagdalenaTulkens, PaulIsmail, SherineTwigg, MichaelIvory, MatthewTzaphlidou, MargarethJalal, ZahraaUm, IreneJamie, KimberlyUrra, José MiguelJanke, KristinVaismoradi, MojtabaJohnson, Kalin L.Vida, YolandaJordan, SueVosper, HelenKanekiyo, TakahisaWang, ZhijunKarpiński, Tomasz M.Ward, NicolaKaruppagounder, VengadeshprabhuWeldon, SharonKastaun, SabrinaWietholter, Jon P.Keers, RichardWitry, MatthewKennedy, Mary-ClaireWu, TuoqiKnopf, HildtraudXiong, ZhaohuiKnouse, Laura E.Yamamura, ShigeoKoh, UyenYancey, AbigailKok, GerjoYap, KevinKosari, SamYau, Wai PingKovvasu, SuryaYeung, EugeneKoyama, YuYunusa, IsmaeelKrause, Jane E.Zgarrick, DavidKreling, David H.Zhang, YuanKreuzpointner, LudwigZhen, Yap KaiKubota, RieZimmerman, Kanecia ObieKuiper, Ruth AnneZineldin, MosadKumar, NitinZou, PingLampkin, Stacie


